# Clinical Society of the New York Polyclinic Medical School and Hospital—Meeting October 6, 1902

**Published:** 1903-06

**Authors:** 


### Clinical Society of the New York Polyclinic Medical School and Hospital—Meeting October 6, 1902.

### FRACTURE OF THE PATELLA.

The President, Dr. Alexander Lyle, in the chair.

Dr. J. A. Bodine exhibited a patient with the following history: Six weeks ago this patient sustained a fracture of the patella. As in the great majority of cases in which the line of fracture is transverse in direction, the cause was a sudden, involuntary contraction of the quadriceps muscle, while the leg was in opposed flexion. The fact that he is already a cripple in the other leg demanded that bony union with good functional results be obtained, and a stringent financial condition urged the accomplishment of this in the shortest space of time possible. I present him to you tonight ready to resume his occupation with a perfectly functionating leg.

In my opinion, all cases of fracture of the patella should be treated by open suture, as was done in this case, provided it is done by a trained surgeon. Primary union is absolutely essential. The most difficult accomplishment in surgery today is not mechanical skill in operating, but comparative cleanliness in technique, with minimized traumatism to tissues. This attainment is only relatively possible to the trained operator, and absolutely impossible to the general practitioner who occasionally operates.

The treatment of fractured patella by mechanical means, splints, strapping, subcutaneous suture, etc., is irrational and wrong fundamentally. If the line of fracture extends laterally into the expansion of the patella capsule, with wide separation of the fragments, in but very few cases will any method other than that by open suture produce a perfectly functionating leg. The reason is simple. When the patella fractured by muscular contraction comes over a bent knee the stretched capsule, when it ruptures, projects beyond the edges of the fragments and, as pointed out by Macewen years ago, falls between the broken fragments. The interposition of this ever present fibro-periosteal fringe is an obvious obstruction to bony contact or bony union, and must be removed by bone operation. Again, blood from the broken bones and torn capsule fills the intervening space and distends the joint, if this is opened, with clotted blood. What becomes of this aseptic blood-clot? On the same great proven principle of Schede's aseptic blood-clot in bone surgery, it becomes more or less organized into living, fibrous tissue, fixing the joint and surrounding tissues in a condition of more or less rigidity and ankylosis. Obviously the thing to do is to remove both the blood-clot and the capsular fringe. The wonder is not that, by the old method, the joint is more or less impaired, but that it functionates at all. Yet, I repeat that if one is not a trained surgeon, it is better to accept this result; at least it will not kill the patient from sepsis.

The simplicity and ease of accomplishment which characterizes the result here presented to you is remarkable. A quarter of a one per cent solution of cocaine was injected before making a transverse skin incision. As the patella lies subcutaneously and its fibrous periosteal capsule is already ruptured from the accident, this skin incision admits one directly to the field of operation. Nothing remains to be done but to wash away the blood clots, remove the interposed fringe of capsule, and suture, not the bone, but the capsular rent, as well as any rent extending into the lateral capsular expansion. In a great many cases, contrary to text-book teaching of the present day, the knee-joint is not opened by the accident nor invaded by the operation. This is due to the fact that the closed synovial sac of the joint is frequently reflected from the posterior surface of the patella at or near the median line. Consequently, any fracture below the level of this line of reflection does not enter the general articular cavity. This condition of extra articular safety I have encountered several times.

The skin incision being closed, if the work has been done aseptically, there is no swelling, no pain, no fever. Beginning about the third week, carefully graduated passive motion, with daily massage is inaugurated. At the end of the sixth week a perfect cure will result, as exemplified by this case presented tonight.

Dr. W. R. Townsend opened the discussion by saying that it is unusual to find a surgeon who never encounters sepsis as a result of his work. All surgeons should be clean, and practically speaking, all are in a majority of cases, but surgeons have not reached the point of perfection asepsis. Especially in serious cases, such as opening the knee-joint, if an operator can avoid suppuration, so much the better, but there as so many possibilities of infection that he would hesitate before undertaking this operation. The growth of small hospitals throughout the country is very rapid, and he thought that the surgery of the future should be done in such places. He did not believe that a similar result can be counted on in every case of fractured patella by the method advocated by Dr. Bodine, and he said that he had seen some very good results by the old method, and in many instances in which it failed the fault was not due so much to the method as to the operator. Bony union is always better than fibrous union; yet he would hesitate very much in saying that every fractured patella should be treated by the open method.

Dr. W. H. Luckett said that if every case that was operated on turned out as successfully as Dr. Bodine's case did, he would be inclined to advocate the open method in all instances, but, unfortunately, all surgeons are not aseptic. He did not agree with Dr. Bodine that blood in a synovial cavity goes through the same process of organization as blood in a bone cavity. Here the blood is in contact with a very rapidly absorbing surface, but more often blood exuded into an articular cavity will be absorbed rather than become organized. The most important treatment in connection with fracture of the patella is massage, applied particularly to the quadriceps muscles, and this should be forcible enough to allow relaxation of that muscle and juxtaposition of the fractured fragments of the patella. The bone must be retained in place by straps and splints. The massage should be done properly by one who thoroughly understands the principles involved. This method was first introduced, he thought, by Dr. Howard Lilienthal.

Dr. W. C. Cilday said that he had a patient who was treated by the old school method, and the result was most unsatisfactory. He had been with Dr. Bodine when he operated on the patient presented this evening and having seen the result, he should hereafter treat all his cases in that manner.

Dr. Bodine closed the discussion. He said in reply to Dr. Luckett that he did not mean to imply that every hemorrhage into the knee-joint becomes organized on the principle of Shede's moist blood-clot, but he was quite sure that this hemorrhage into the cavity of the knee-joint and into the tissues surrounding the knee is a cause, in part at least, of the many cases of rigiditv or partial ankylosis in fracture of the patella; and, furthermore, he was sure this rigidity is caused by a more or less complete organization of the blood-clot into the living tissues. He had never seen the statement emphasized that in fracture of the patella the general articular cavity is not opened. This statement was based on the fact that twice in his experience he had encountered a condition of an intact synovial membrane beneath the broken fragments. It is a well-known anatomical fact that the reflection of the synovial membrane on the posterior surface of the patella as high as its middle posterior line, frequently occurs, so that all fractures of this bone occurring below the level of this line of reflection would not involve the general synovial cavity of the joint. A knee-joint filled with blood-clot, even though there is no external wound, may become infected through the medium of the circulation. He believes that the frequent occurrence of synovial osteomyelitis is sufficient proof of this statement. He had known of occurrences of violent infection within the knee-joint following fracture of the patella when no operation had been attempted. If this infection were due to an external wound in the surface he was unable to find the point of entrance.

THREE CASES OF TETANUS.

Dr. Luckett also reported three cases of tetanus. The first patient was admitted into the Harlem Hospital with a blank-cartridge wound of the palm of the left hand. Within ten hours he had developed all the symtoms of a most pronounced case of tetanus. Treatment was by the usual method—potassium bromide, chloral hydrate, hot baths, etc. The second case was that of a wound in the palm of the hand from a blank cartridge. Symptoms of tetanus developed on the eighth day—opisthotonos, lockjaw, and local tetanus of the left hand. Treated by injections of the New York Board of Health anti-tetanic serum in extraordinarily large quantities between the third and fourth lumbar vertebræ into the subarachnoid cavity. After the cerebro-spinal fluid had been withdrawn the patient received an injection daily for about 10 days, getting from 8 to 10 cc. at each injection, from 10 to 45 minims of cerebro-spinal fluid being withdrawn previous to each injection. From the very start his symptoms improved. The patient was discharged from the hospital cured. The speaker believed this to be the first time that this method was used in this country.

The third case was a more pronounced type of tetanus than the preceding one. It occurred in a boy, nine years old, who jumped over a garden fence and cut his left wrist on the neck of a broken bottle. The wound was sutured at one of the hospitals. On the sixth day the boy developed stiffness of the muscles of the neck, of the abdomen, and of the jaw, in the order given. He was admitted into the Harlem Hospital, the wound was opened, several particles of dirt were removed therefrom, and the wound was dressed. Very marked symptoms of tetanus were present. This boy, although in appearance much worse than the preceding one, recovered more rapidly under treatment, only five injections being necessary. This was attributed to the fact that preceding each injection, all of the cerebro-spinal fluid possible was removed, as much as 560 minims in five days. The patient was discharged, cured, about two and one-half weeks after admission. The reason for withdrawing this fluid is on account of its great toxity, it being much more toxic than the blood of a tetanus patient. These patients, except the first one, received no internal medication whatever.

